# Lyapunov estimation for high-speed demodulation in multifrequency atomic force microscopy

**DOI:** 10.3762/bjnano.9.47

**Published:** 2018-02-08

**Authors:** David M Harcombe, Michael G Ruppert, Michael R P Ragazzon, Andrew J Fleming

**Affiliations:** 1 School of Electrical Engineering and Computing, The University of Newcastle, Callaghan, NSW, 2308, Australia; 2 Department of Engineering Cybernetics, NTNU, Norwegian University of Science and Technology, Trondheim, Norway

**Keywords:** atomic force microscopy (AFM), demodulation, digital signal processing, field-programmable gate array (FPGA), high-speed, Lyapunov filter, multifrequency

## Abstract

An important issue in the emerging field of multifrequency atomic force microscopy (MF-AFM) is the accurate and fast demodulation of the cantilever-tip deflection signal. As this signal consists of multiple frequency components and noise processes, a lock-in amplifier is typically employed for its narrowband response. However, this demodulator suffers inherent bandwidth limitations as high-frequency mixing products must be filtered out and several must be operated in parallel. Many MF-AFM methods require amplitude and phase demodulation at multiple frequencies of interest, enabling both *z*-axis feedback and phase contrast imaging to be achieved. This article proposes a model-based multifrequency Lyapunov filter implemented on a field-programmable gate array (FPGA) for high-speed MF-AFM demodulation. System descriptions and simulations are verified by experimental results demonstrating high tracking bandwidths, strong off-mode rejection and minor sensitivity to cross-coupling effects. Additionally, a five-frequency system operating at 3.5 MHz is implemented for higher harmonic amplitude and phase imaging up to 1 MHz.

## Introduction

Atomic force microscopy (AFM) [1] has been integral in the field of nanoscale engineering since its invention in 1986 by Binnig et al. By sensing microcantilever tip–sample interactions [2], atomic scale resolution imaging is achieved, which far exceeds the optical diffraction limit. An image generated by constant-force topography AFM depends entirely on its feedback control loop. The composition of a sample is visualized in three-dimensions by plotting the control signal against the lateral scan trajectories of the nanopositioner.

In static-mode AFM (contact mode), the control loop attempts to maintain a constant contact force [3]. Where as in dynamic modes, for example intermittent-contact constant-amplitude AFM [4], the control loop acts to maintain a constant cantilever oscillation amplitude. This is achieved by feeding back the demodulated fundamental frequency present in the deflection signal. In intermittent-contact mode AFM [5], the tapping amplitude is chosen such that only gentle tip–sample interactions occur. This is particularly suitable for studying biological samples, allowing for biophysical processes to be studied [6–8].

Multifrequency AFM (MF-AFM) methods allow for the study of tip–sample interactions occurring at multiple frequencies [9]. This extends imaging information beyond the topography to a range of nanomechanical properties including sample stiffness, elasticity and adhesiveness [10]. The acquisition of these observables requires tracking the amplitude and phase of additional frequencies of interest. These include higher harmonics of the fundamental frequency [11], higher flexural eigenmodes [12] and intermodulation products [13]. Higher harmonic methods have demonstrated the ability to image relatively large biological objects, such as cells [14–15], while bimodal AFM has successfully imaged properties of protein complexes [16]. Intermodulation AFM is a novel extension to the bimodal method that focuses on the mixing products of a slightly below and above resonance bimodal drive. It has been shown to achieve increased image contrast [17] and lead to further insights into nanomechanical properties [18]. Regardless of which particular MF-AFM method is employed, they each require the demodulation of amplitude and phase to form observables for the characterization of nanomechanical properties.

**Figure 1:**
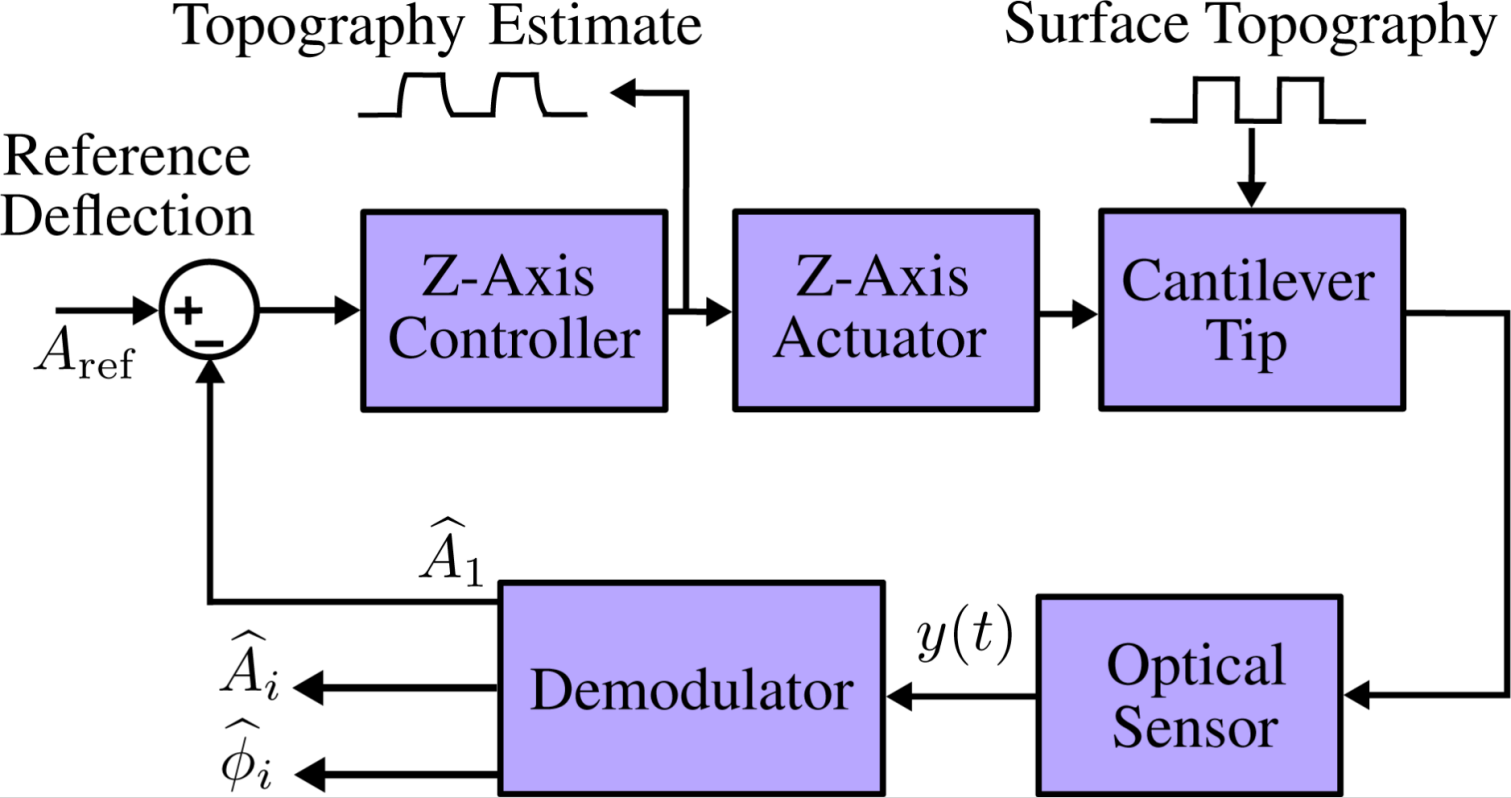
Block diagram of the multifrequency AFM control loop.

Due to the large bandwidth requirements of tracking high frequencies in MF-AFM, every component of the *z*-axis feedback loop detailed in Figure 1 needs to be optimized for speed. This includes the lateral and vertical nanopositioner for each axis (*x*, *y* and *z*), cantilever, vertical feedback controller and demodulator. In this article, the demodulator component is improved with respect to its key performance metrics: tracking bandwidth, sensitivity to other frequency components, and implementation complexity. Tracking bandwidth is defined as the point in a frequency response where the output of the demodulator drops by −3 dB with respect to the input modulating signal. Off-mode rejection (OMR) is a term that describes the attenuation of unwanted frequencies present in the input signal, which lie outside the modeled carrier of interest.

It has been shown that conventional high-speed demodulation techniques are incompatible with MF-AFM, due to the lack of sensitivity to multiple frequency components [19]. These include the peak detector [20], peak-hold [21] and RMS-to-DC [22] conversion methods. A typical MF-AFM demodulator employs multiple lock-in amplifiers (LIA) in parallel, as each provides an accurate estimation of a particular frequency component. However, low-pass filters are employed to diminish mixing products, which severely limits the demodulator bandwidth [23].

Motivated by improving MF-AFM demodulation capabilities, previous work by the authors includes a multifrequency Kalman filter [24]. It was shown to outperform a commercially available LIA in terms of both tracking bandwidth and noise performance. However, a major disadvantage of the Kalman filter is the large computational expense of each additional frequency modeled. This reduces its realizable performance through limitations of the sample rate. An estimator in the form of a Lyapunov filter [25] was demonstrated to perform similarly to the Kalman filter [26]. However, the Lyapunov filter complexity scales significantly better than the Kalman filter when multiple frequencies are modeled [27].

This article extends previous work by providing a thorough performance analysis of the multifrequency Lyapunov filter in terms of tracking bandwidth, off-mode rejection and cross-coupling effects. In addition, MF-AFM demodulation is demonstrated by performing higher harmonic imaging with amplitude and phase on both a stiff and compliant sample.

## Lyapunov Filter

### System modeling

A single-frequency cantilever deflection signal is modeled as a sine wave with carrier frequency *f*_c_, time-varying amplitude *A*(*t*) and phase 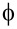
(*t*) of the form

[1]
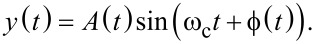


For readability, explicit dependencies on time of the amplitude *A*(*t*) and phase 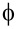
(*t*) are dropped from this point onward. By extension, a deflection signal consisting of multiple frequencies is modeled as

[2]
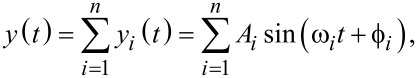


where *i* = 1, 2, …, *n* denotes the *i*-th modeled frequency. Through the double-angle trigonometric identity, this model is linearly parameterizable such that vector pairs within the state vector **x** = 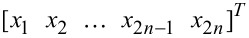 represent quadrature and in-phase components of each particular modeled frequency. That is, each individual sine wave is represented by

[3]
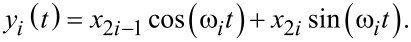


Based on the parametrization of the signals in Equation 3, the time-varying amplitude and phase of a particular frequency is recovered by

[4]
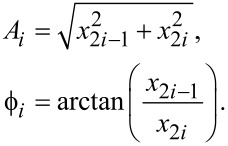


### Filter description

**Figure 2:**
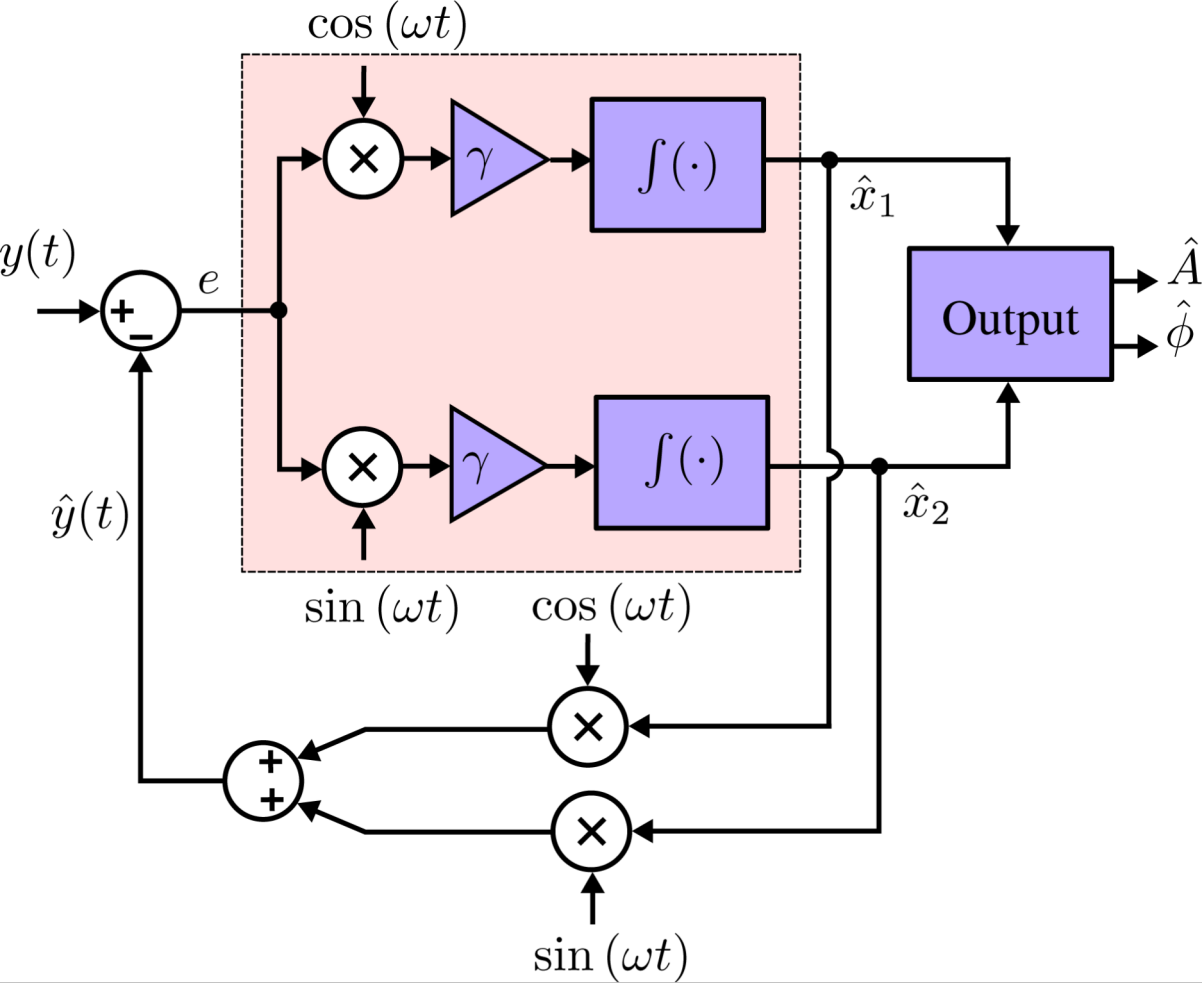
Block diagram of a single-frequency Lyapunov filter. The pink shaded area highlights the calculation that can be done in parallel for multiple frequencies.

The Lyapunov filter [28] is implemented as a linear observer as shown in Figure 2. A key property of the filter is exponential convergence of the estimated states [29], with the tunable loop gain constant γ governing the speed of convergence. The filter is shown to have a negative feedback loop in which integral action regulates the error. By feeding back an estimate of the input signal obtained from the parameterized states in the form of Equation 3, an error signal is generated. Regulation of this error through feedback leads to the much desired suppression of the *2f*_c_ mixing components.

The update law for the single-frequency Lyapunov filter [28] can be extended to a multifrequency form, resulting in

[5]
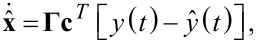


[6]
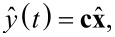


where

[7]
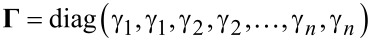


and

[8]
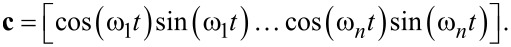


In this form, 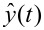 represents the estimated output and the amplitude *A_i_* and phase 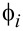 are available by applying Equation 4 to each quadrature and in-phase pair of 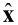. A key requirement to ensure exponential convergence of 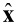 to **x**, is to guarantee that *c* is persistently excited [29]. Convergence is shown for the single-frequency filter in [28], and can easily be extended for the multifrequency case. Furthermore, exponential convergence of 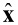 means that 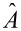 and 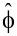 also converge.

## Results and Discussion

### Hardware

The Lyapunov filter was implemented on a high-speed FPGA to achieve the necessary sample rate for accessing higher harmonics during imaging. A Xilinx Kintex-7 KC705 evaluation board (model: XC7K325T) paired with a DC-coupled high-speed 4DSP input/output (I/O) card (model: FMC151) was utilized. The FPGA clock is synchronized with the high-speed I/O card at 250 MHz. The I/O card has a two-channel 14-bit analog-to-digital converter (ADC) and a two-channel 16-bit digital-to-analog converter (DAC), which sample at 250 MHz and 800 MHz, respectively.

### Implementation

Figure 2 shows the block diagram of a single-frequency Lyapunov filter (SF-LYAP). Here, the digital components required for FPGA implementation can be seen: multipliers, adders, registers, sample rate control for feedback and a programmable direct digital synthesizer (DDS). The DDS generates the sine (in-phase) and cosine (quadrature) signals required to model carrier frequencies. It may be tuned through control of its frequency word which is calculated by

[9]
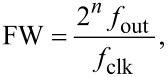


where *f*_out_ is the desired output frequency, FW represents the binary word required to program the DDS, *n* is the length of FW and *f*_clk_ is the speed of the FPGA board.

A SF-LYAP was successfully implemented at a sampling rate of *f*_s_ = 5 MHz. As stability is of priority, the chosen data representation is floating point in the standard IEEE 754 format. The integration method used is backward Euler, as this ensures stability when γ is large [30]. The output equation (Equation 4) is realized with the Xilinx Coordinate Rotation Digital Computer (CORDIC), set to a 16-bit configuration such that the amplitude and phase are formatted for the I/O card. The carrier frequency *f*_c_, γ and any necessary output gains for amplifying very small signals during imaging are tunable in real-time using the Xilinx Virtual Input Ouput (VIO) tool.

**Figure 3:**
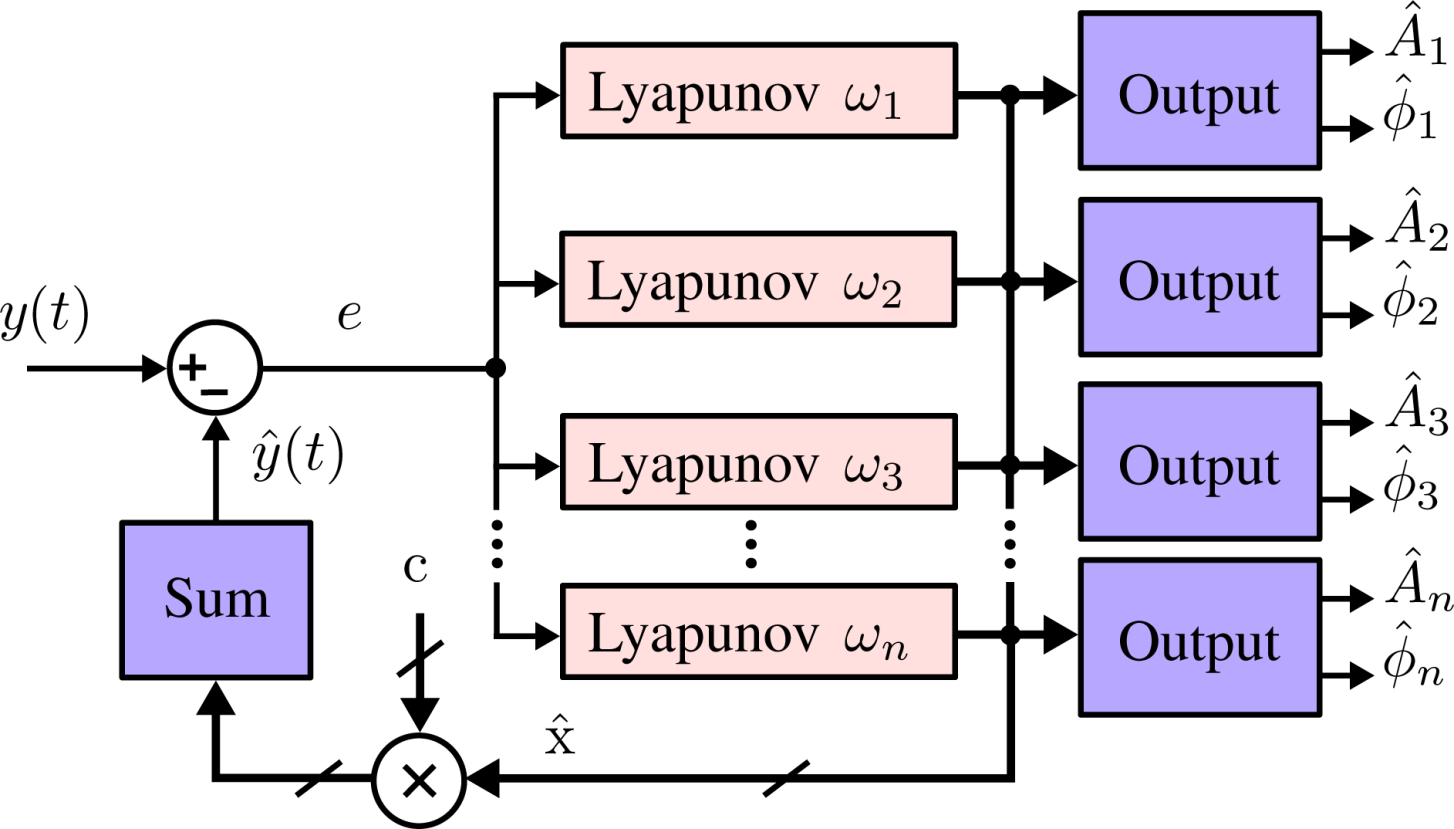
Block diagram of a multifrequency Lyapunov filter.

In Figure 3, the block diagram of the implemented multifrequency Lyapunov filter (MF-LYAP) is shown. Here, it can be seen that an MF-LYAP involves several SF-LYAPs running in parallel. Channel cross-coupling occurs in the combined output feedback as dictated by the output equation (Equation 6). The Lyapunov filters timing constraints for a five-frequency system result in a maximum sampling rate of *f*_s_ = 3.5 MHz. This is a large improvement over the multifrequency Kalman filter [24], which was 1.5 MHz for a three-frequency system. The Kalman filter equations [24] can be shown to have a complexity of 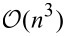, while that of the Lyapunov filter is 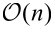 for *n* modeled frequencies. This stark difference in complexity arises from the computations required for the Kalman gain and covariance matrix update.

### Experimental setup

A LIA (Zürich Instruments HF2LI) was used in-conjunction with a laboratory function generator (Agilent 33521A waveform generator) to experimentally verify the performance of the implemented Lyapunov filter. These investigations include a frequency response experiment to measure the tracking bandwidth and channel cross-coupling. Additionally, off-mode rejection of channels in both high-speed and slow configurations was explored through a carrier sweep.

### Tracking bandwidth

**Figure 4:**
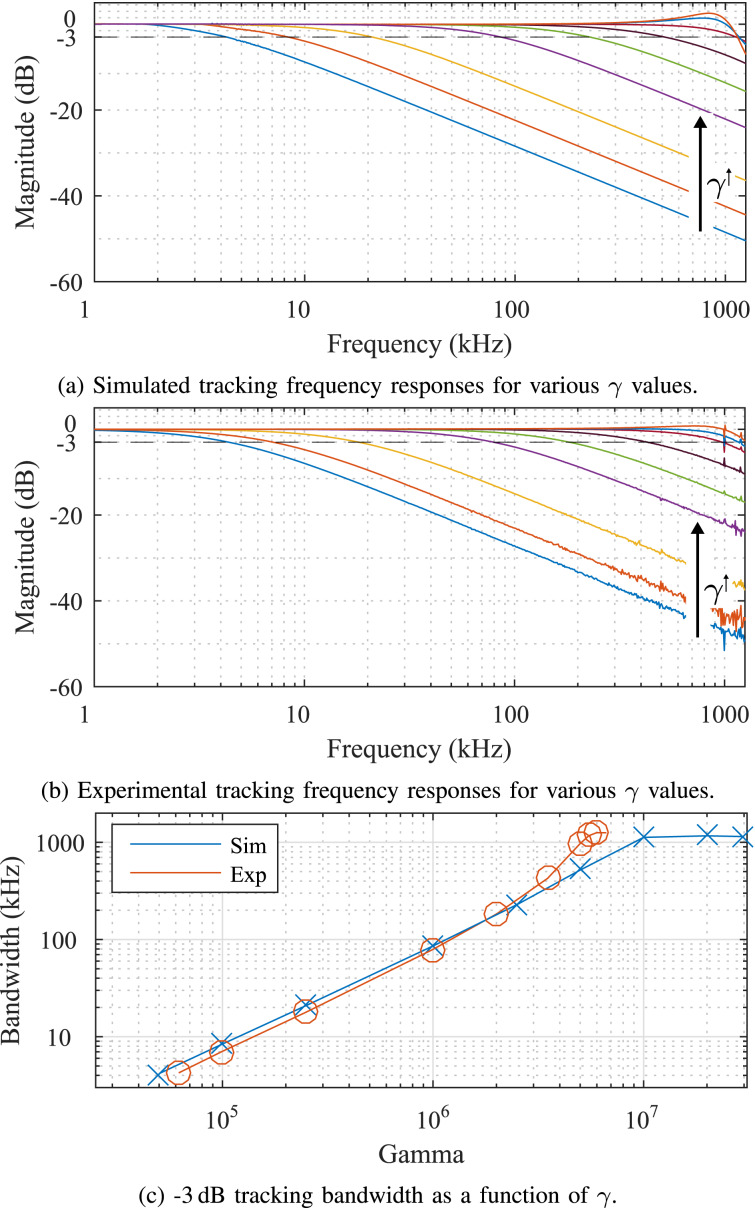
(a) Simulated and (b) experimentally obtained frequency responses of the Lyapunov filter with a carrier frequency of 1 MHz and varying tracking bandwidths, as indicated by the increasing γ values. Results in (c) show the −3 dB tracking bandwidth of each system as a function of γ.

The tracking bandwidth of the Lyapunov filter was characterized through frequency responses from both a simulated and experimentally implemented system. For each frequency response, the modulating signal *A*(*t*) in Equation 1 was swept from DC to 1.5 MHz while the carrier frequency was held constant. The tracking bandwidth experiment examines the relationship between the −3 dB point and γ for a 1 MHz carrier frequency and γ values ranging between 5 × 10^4^ and 1 × 10^7^. Figure 4 shows the results of (a) simulated and (b) experimental tracking frequency responses, where it can be seen that the two systems match closely. The similarity was achieved by maintaining a consistent sample rate and integration method for both simulation and experimental implementation. In Figure 4c, the simulated and experimental −3 dB points are shown as a function of the tunable loop constant γ. For both systems, the tracking bandwidth approaches the carrier frequency *f*_c_.

**Figure 5:**
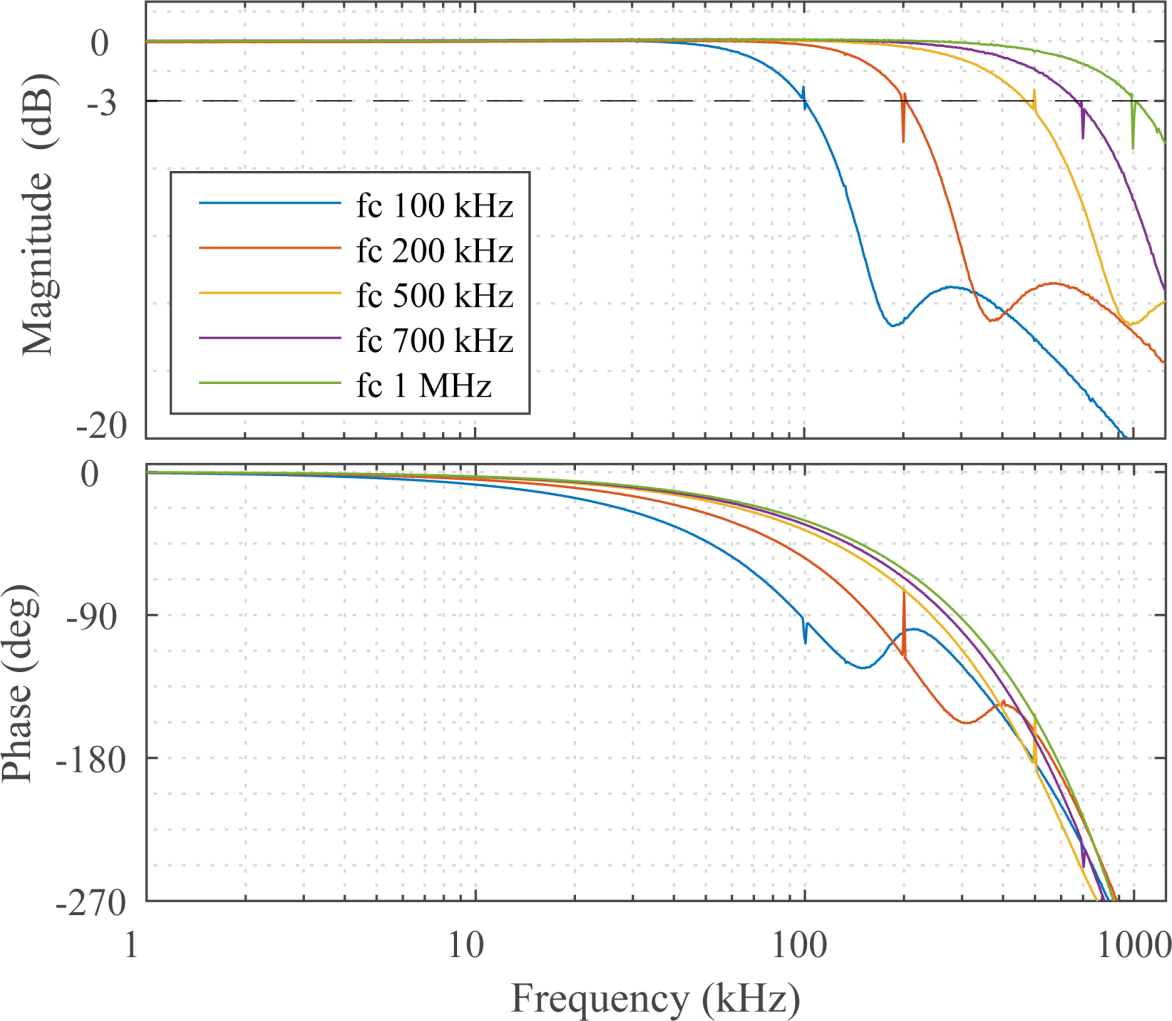
Experimental frequency responses from a single-frequency Lyapunov filter with five carrier frequencies tuned to a −3 dB tracking bandwidth of *f*_c_.

Figure 5 demonstrates several cases in which the Lyapunov filter is achieving a high tracking bandwidth of *f*_c_, the equivalent of single cycle tracking. This was achieved for the five carrier frequencies 100 kHz, 200 kHz, 500 kHz, 700 kHz and 1 MHz with γ values of 1.2 × 10^6^, 2.2 × 10^6^, 3.7 × 10^6^, 4.4 × 10^6^ and 5.1 × 10^6^, respectively.

### Cross-coupling

The effect of channel cross-coupling on the tracking bandwidth was examined for both a simulated and experimentally implemented system. For simplicity, cross-coupling was demonstrated with a two-frequency MF-LYAP wherein the modeled carrier frequencies are 100 kHz and 500 kHz for channels 1 and 2, respectively. Each channel is considered for two fixed tracking bandwidth settings, low (1 kHz) and high (50% of *f*_c_), while the other channel is increased in speed.

**Figure 6:**
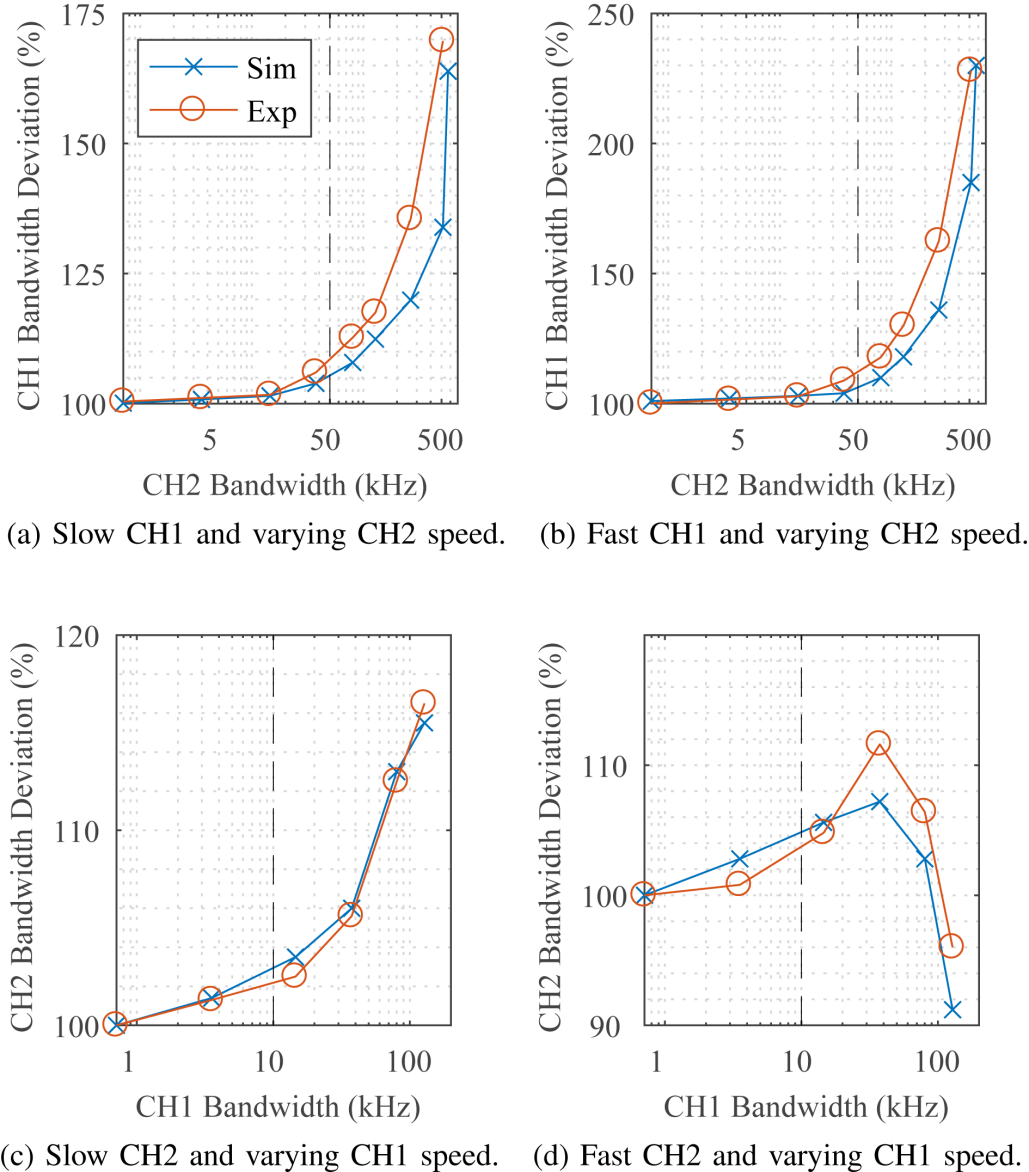
The effect of channel cross-coupling on tracking bandwidth for a system based on two frequencies. The effect of the speed of channel 2 on channel 1 set to (a) slow (1 kHz) and (b) high-speed (50% *f*_c_). Similarly, the effect of the speed of channel 1 on a (c) slow and (d) high-speed channel 2. The dashed line in each plot represents a speed of 10% of *f*_c_.

Figure 6a–c shows that the tracking bandwidth of a channel will increase from its original setting as the other channel is tuned faster. Conversely, Figure 6d shows channel 2 slowing down as channel 1 is increased in speed. This is explained by the fact that channel 2 is set to a tracking bandwidth of 250 kHz (50% *f*_c_), which is higher than the maximum obtainable speed of channel 1. Throughout this investigation, the simulation and experimental results agree. The results show that cross-coupling effects are more pronounced in low-speed channels. They are, however, negligible if tracking bandwidths of channels remain below 10% of *f*_c_.

### Off-mode rejection

The off-mode rejection of the multifrequency Lyapunov filter was analyzed by performing a single-tone sine sweep on the input signal and recording the demodulated amplitude magnitude of each channel. For each frequency response, the carrier frequency *f*_c_ in Equation 1 was swept from DC to 1.25 MHz with a constant amplitude *A*. This experiment used a five-frequency MF-LYAP with channels set to carriers of 100 kHz, 200 kHz, 500 kHz, 700 kHz and 1 MHz for both a simulated and experimentally implemented system.

**Figure 7:**
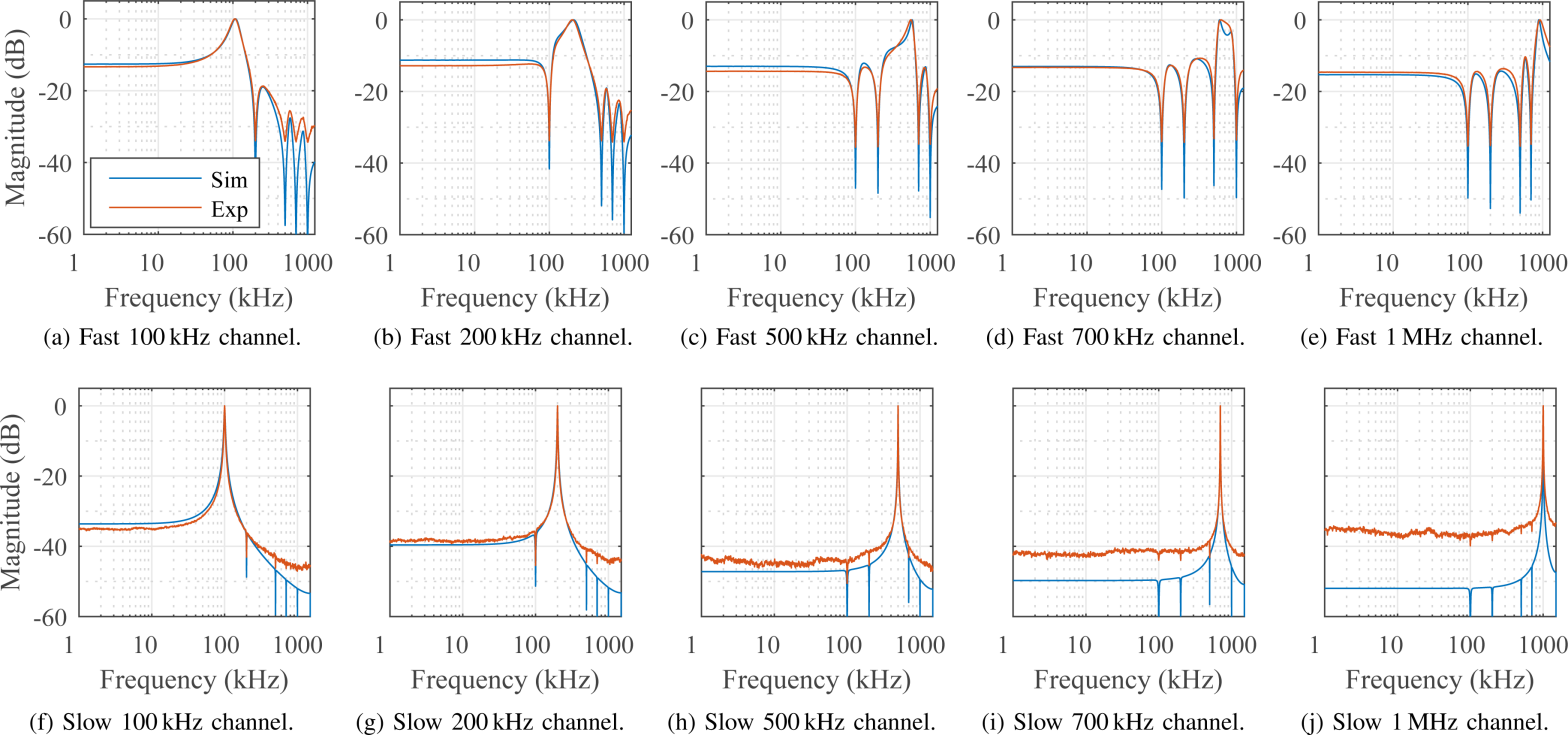
Experimental and simulated off-mode rejection frequency responses for both (a–e) fast demodulator (10% of *f*_c_) and (f–j) slow demodulator (1 kHz) bandwidth settings.

Figure 7a–e shows off-mode rejection for a fast (10% of *f*_c_) tracking bandwidth setting. For each channel, a full recovery (0 dB) of the signal can be seen to occur at its modeled carrier frequency, as expected. There is strong off-mode rejection occurring at the other modeled carrier frequencies, due to output feedback cross-coupling sharing state information between channels. Figure 7f–j demonstrates off-mode rejection for a slow (1 kHz) tracking bandwidth setting. Here, the narrowband response is a direct result of the reduced γ*_i_* values of each channel. This causes a less distinct, but still visible, modeled off-mode rejection at the other carrier frequencies. It can be seen that the slower system achieves greater off-mode rejection outside of the modeled frequencies than the fast system. Again, a similar performance between the simulated and experimental results can be observed. The less distinct off-mode rejection in the experimental results compared to the simulations is due to a finite DC offset from the DAC. This precludes the direct measurement of signals smaller than this value.

### AFM imaging

#### Imaging setup

The Lyapunov filter as a multifrequency AFM demodulator was validated through a series of imaging experiments where it is compared side-by-side to a lock-in amplifier. To ensure a fair comparison, the demodulators were tuned to the same tracking bandwidth in both experiments. This is required as the noise performance has been shown to be a function of the tracking bandwidth [19]. The lock-in amplifier is the state-of-the-art multifrequency method due to its strong off-mode rejection, however it can not achieve the same speed as the Lyapunov filter due to post-mixing filtering [19]. As the high-speed superiority of the Lyapunov filter is well established, it is compared to the lock-in amplifier in a low-speed environment.

Using an NT-MDT NTEGRA AFM, amplitude and phase higher harmonic imaging was performed with a NT-MDT NSG01 and Bruker DMASP cantilever. These cantilevers were found to have fundamental resonance frequencies of 168.8 kHz and 46.1 kHz, respectively. The samples used are a *z*-calibration grating (NT-MDT TGZ3) with periodic height features of approx. 500 nm and a blend of polystyrene (PS) and polyolefin elastomer (LDPE) available from Bruker (PS-LDPE-12M). Due to the different elastic moduli of the PS and LPDE regions, the sample is widely used for qualitative imaging the material contrast.

#### Imaging a TGZ3 calibration grating

**Figure 8:**
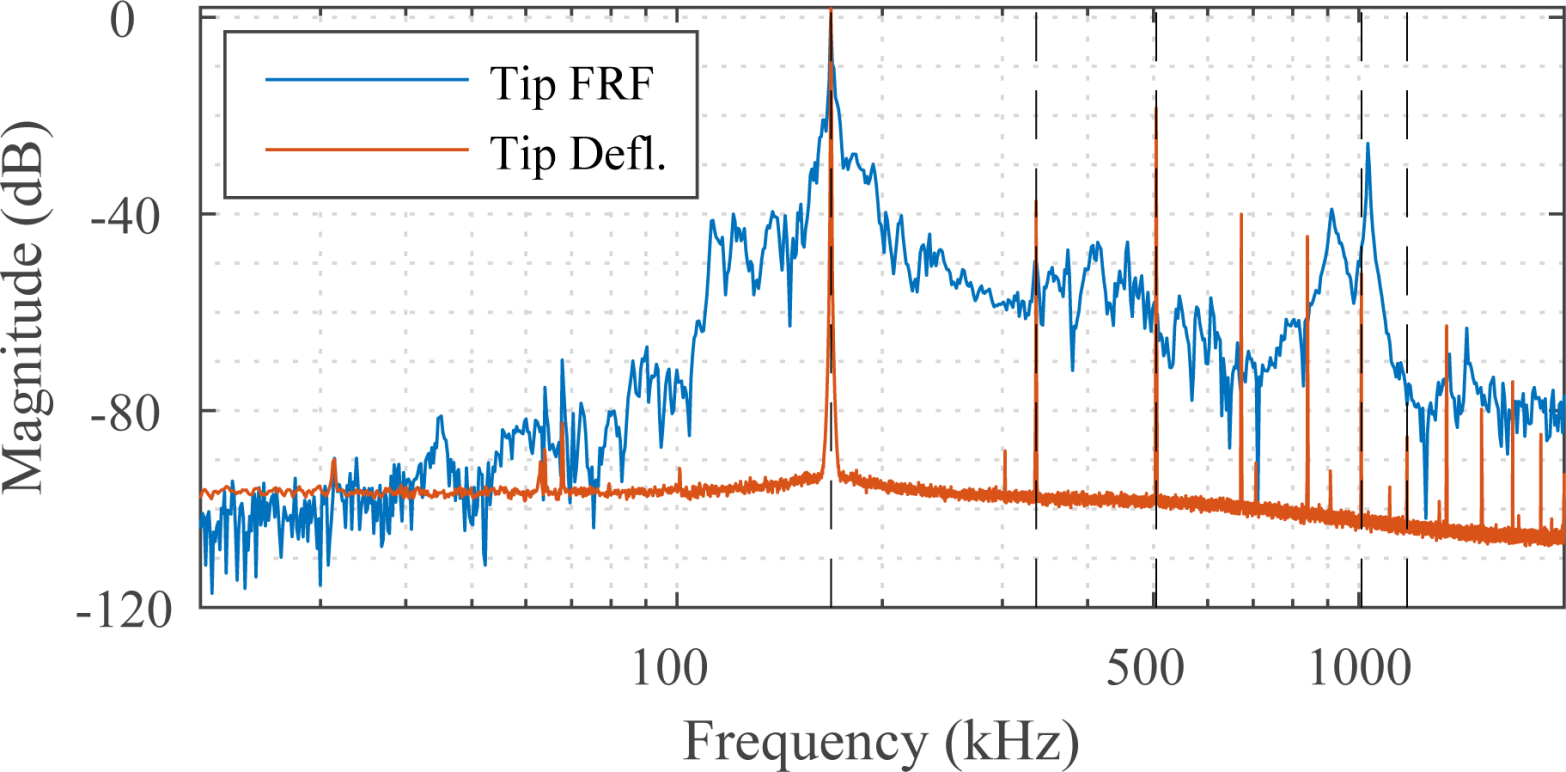
Experimental frequency response of the NSG01 cantilever in free air and the power spectrum of the tip deflection signal in contact with the sample. MF-AFM higher harmonic images obtained with this deflection signal are shown in Figure 9.

Higher-harmonic amplitude images with the first, second, third, sixth and seventh harmonics were obtained by the MF-LYAP and multiple parallel LIAs. The frequency response of the NSG01 cantilever in free air and the power spectrum of its deflection signal during contact are shown in Figure 8. Here, the fundamental and second resonance frequencies can be seen in the cantilever frequency response. The deflection signal spectrum shows additional higher harmonics and minor intermodulation products are present. These are due to non-linear atomic forces exciting the cantilever during contact.

**Figure 9:**
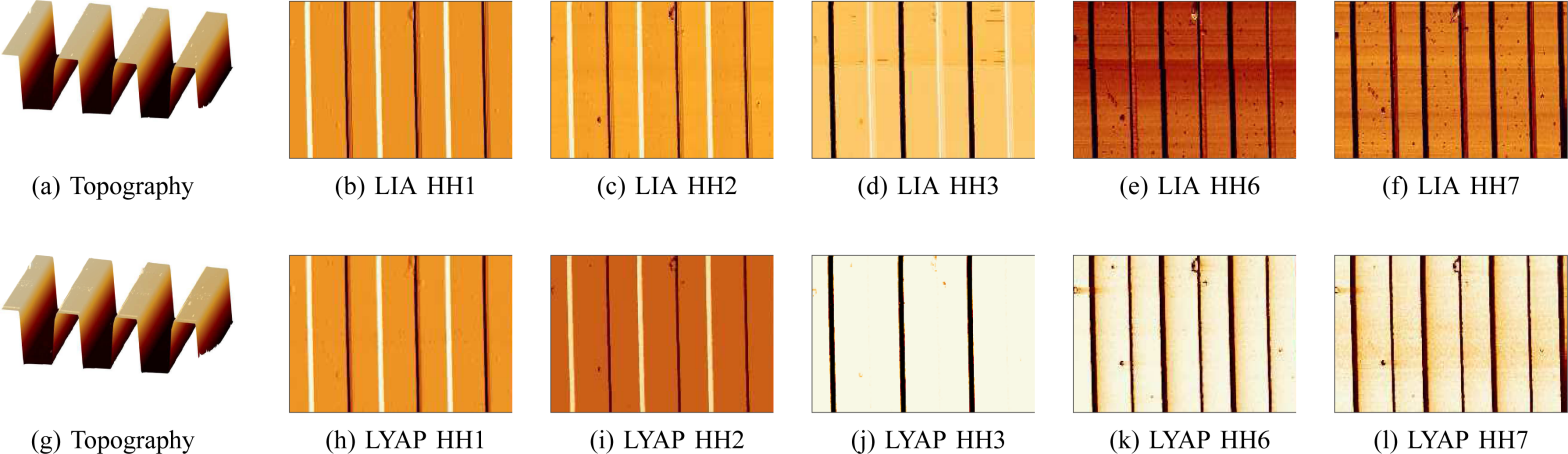
Higher harmonic (amplitude) AFM imaging with a (a–f) lock-in amplifier and (g–l) multifrequency Lyapunov filter on a TGZ3 calibration standard.

Amplitude imaging results are shown in Figure 9. As the sixth and seventh harmonics are closely spaced to the second resonance frequency of the cantilever, they provide an increased signal-to-noise ratio. The MF-LYAP can be seen to perform comparably to the LIA when tuned to similar measurement bandwidth settings. When imaging with higher harmonics, the off-mode rejection of each channel was tuned to suppress the large fundamental frequency.

#### Imaging a PS/LPDE calibration grating

**Figure 10:**
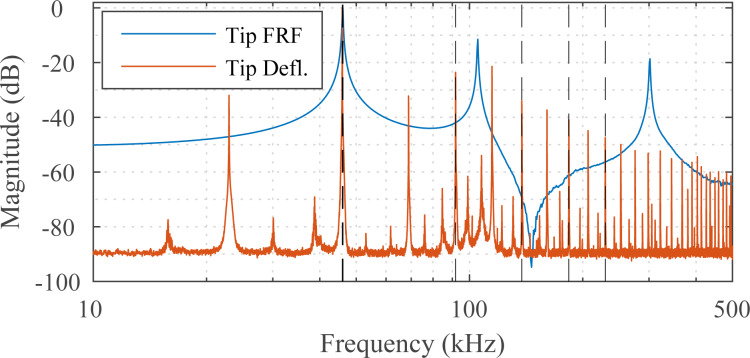
Experimental frequency response of the DMASP cantilever in free air and the power spectrum of the tip deflection signal in contact with the sample. MF-AFM higher harmonic images obtained with this deflection signal are shown in Figure 11.

Higher-harmonic phase images were obtained for the first five harmonics of a Bruker DMASP cantilever. The frequency response of the cantilever in free air and the power spectrum of its deflection signal during contact are shown in Figure 10. Here, the fundamental resonance frequency and higher eigenmodes can be seen in the cantilever frequency response. As before, the deflection signal contains additional higher harmonics and intermodulation products due to the non-linear atomic excitation. Note that the DMASP cantilever uses integrated piezoelectric actuation [31], which results in a clean frequency response when compared to the base excited NSG01 as seen in Figure 8.

**Figure 11:**
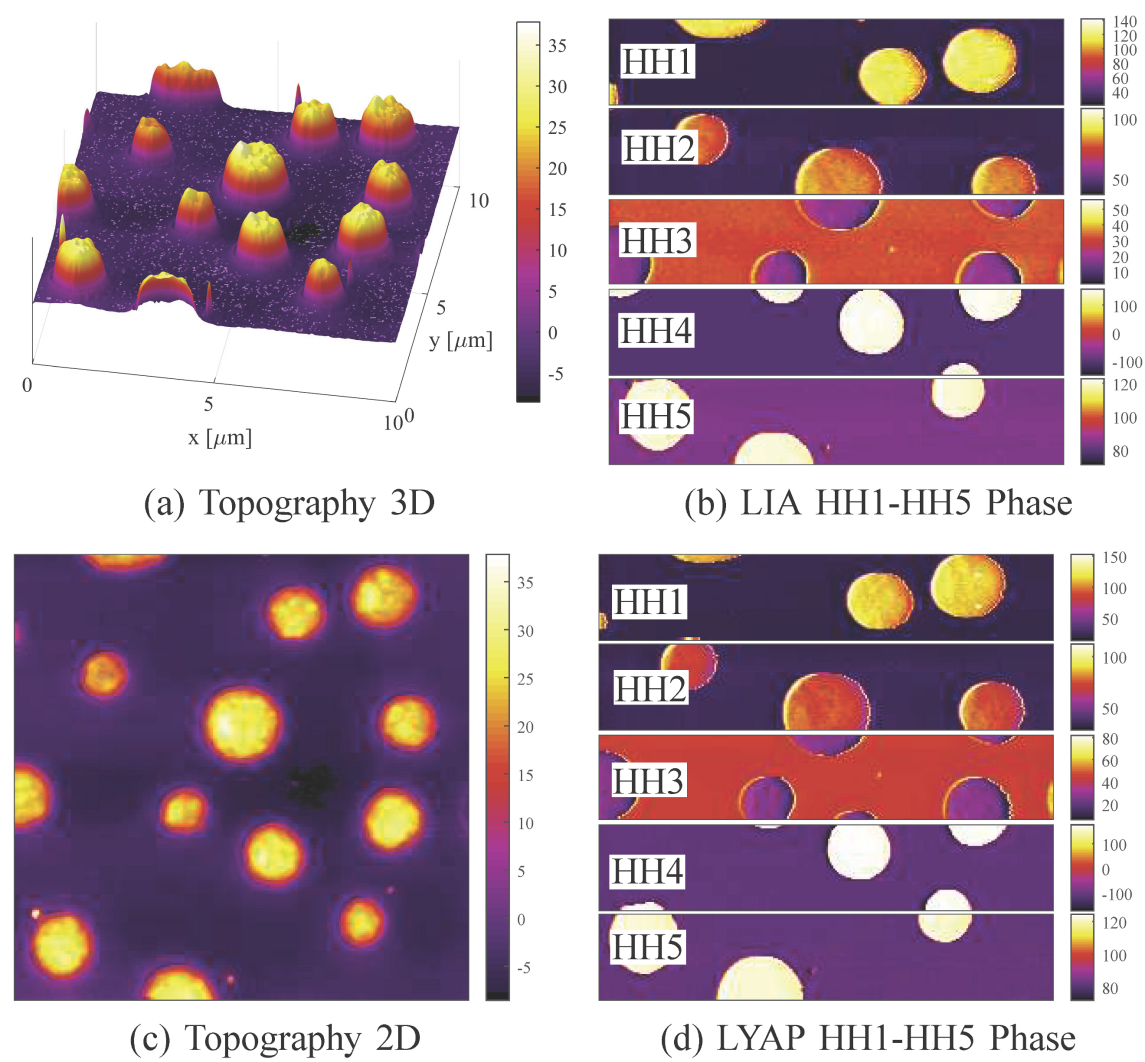
Higher-harmonic AFM imaging performed with the fundamental mode of the DMASP cantilever on a PS/LPDE polymer blend with measurement bandwidths of 1 kHz and 200 Hz for the first and higher harmonics, respectively. (a,c): Topography in nanometers, (b) higher-harmonic phase (°) images taken with parallel LIAs, (d) higher-harmonic phase (°) images taken with the MF-LYAP.

The higher-harmonic phase imaging results are shown in Figure 11. For both demodulators, we see a strong material contrast between the PS and LPDE regions. This was expected from the rich frequency content present in the deflection signal, as visible in Figure 10. We note that the images show particularly strong contrast for the second harmonic, which is due to its proximity to the second mode of the cantilever. This fact is also visible in the increased noise floor around that frequency in the deflection signal. Similarly to the amplitude imaging, the large fundamental frequency contribution required tuning higher harmonics for increased off-mode rejection. For this reason, we tuned the first harmonic demodulators to 1 kHz bandwidth (LIA LPF 1 kHz, LYAP γ = 20 × 10^3^ and the higher harmonics to 200 Hz (LIA LPF 200 Hz, LYAP γ = 2 × 10^3^).

## Conclusion

This article describes a multifrequency Lyapunov filter for high-speed demodulation in MF-AFM. The performance and flexibility of the proposed Lyapunov filter is demonstrated through simulations and experiments. The filter may reach tracking bandwidths up to the modeled carrier frequency, the equivalent of single-cycle tracking. Additionally, the off-mode rejection of the system was found to be controlled by its bandwidth as dictated by the tunable loop constant γ. The relationship between γ and the bandwidth was shown to be linear, up to the modeled carrier frequency. Channel cross-coupling, which occurs due to output feedback, was found to cause distinct rejection of other modeled frequencies during the off-mode rejection experiments. An investigation into this cross-coupling revealed it has negligible effect on the tracking bandwidth of the system.

The multifrequency Lyapunov filter as a flexible, high-speed demodulator was verified through higher harmonic MF-AFM imaging for both amplitude and phase. This demonstrates the filters ability to be used as a demodulator in various MF-AFM techniques involving higher harmonic, higher eigenmode or intermodulation frequency components. In the presented AFM images, the proposed filter performed comparably to a state-of-the-art lock-in amplifier setup. In comparison to the Kalman filter, the Lyapunov filter is similar in terms of speed, off-mode rejection and operation. However, it was found to be significantly easier to implement, which is a priority when considering an extension to multiple frequencies.

## References

[1] Binnig G, Quate C F, Gerber C (1986). Phys Rev Lett.

[2] Rabe U, Janser K, Arnold W (1996). Rev Sci Instrum.

[3] Abramovitch D Y, Andersson S B, Pao L Y, Schitter G (2007). A tutorial on the mechanisms, dynamics and control of atomic force microscopes. 2007 American Control Conference.

[4] García R (2011). Amplitude modulation atomic force microscopy.

[5] Zhong Q, Inniss D, Kjoller K, Elings V B (1993). Surf Sci.

[6] Möller C, Allen M, Elings V, Engel A, Müller D J (1999). Biophys J.

[7] Kodera N, Yamamoto D, Ishikawa R, Ando T (2010). Nature.

[8] Chiaruttini N, Redondo-Morata L, Colom A, Humbert F, Lenz M, Scheuring S, Roux A (2015). Cell.

[9] García R, Herruzo E T (2012). Nat Nanotechnol.

[10] Garcia R, Proksch R (2013). Eur Polym J.

[11] Stark R W, Heckl W M (2003). Rev Sci Instrum.

[12] Martínez N F, Lozano J R, Herruzo E T, Garcia F, Richter C, Sulzbach T, Garcia R (2008). Nanotechnology.

[13] Platz D, Tholén E A, Pesen D, Haviland D B (2008). Appl Phys Lett.

[14] Raman A, Trigueros S, Cartagena A, Stevenson A P Z, Susilo M, Nauman E, Contera S A (2011). Nat Nanotechnol.

[15] Cartagena-Rivera A X, Wang W-H, Geahlen R L, Raman A (2015). Sci Rep.

[16] Herruzo E T, Perrino A P, Garcia R (2014). Nat Commun.

[17] Forchheimer D, Forchheimer R, Haviland D B (2015). Nat Commun.

[18] Thorén P-A, de Wijn A S, Borgani R, Forchheimer D, Haviland D B (2016). Nat Commun.

[19] Ruppert M G, Harcombe D M, Ragazzon M R P, Moheimani S O R, Fleming A J (2017). Beilstein J Nanotechnol.

[20] Ando T (2012). Nanotechnology.

[21] Ando T, Kodera N, Takai E, Maruyama D, Saito K, Toda A (2001). Proc Natl Acad Sci U S A.

[22] Kitchin C, Counts L (1986). RMS to DC Conversion Application Guide.

[23] Ruppert M G, Karvinen K S, Wiggins S L, Moheimani S O R (2016). IEEE Trans Control Syst Technol.

[24] Ruppert M G, Harcombe D M, Moheimani S O R (2016). IEEE/ASME Trans Mechatronics.

[25] Ragazzon M R P, Gravdahl J T, Fleming A J (2016). On Amplitude Estimation for High-Speed Atomic Force Microscopy. 2016 American Control Conference (ACC).

[26] Ruppert M G, Harcombe D M, Ragazzon M R P, Moheimani S O R, Fleming A J (2017). Frequency domain analysis of robust demodulators for high-speed atomic force microscopy. 2017 American Control Conference (ACC).

[27] Harcombe D M, Ruppert M G, Ragazzon M R P, Fleming A J (2017). Higher-harmonic AFM imaging with a high-bandwidth multifrequency Lyapunov filter. 2017 IEEE International Conference on Advanced Intelligent Mechatronics (AIM).

[28] Ragazzon M R P, Ruppert M G, Harcombe D M, Fleming A J, Gravdahl J T (2017). IEEE Trans Control Syst Technol.

[29] Ioannou P A, Sun J (2012). Robust Adaptive Controls.

[30] Cellier F E, Ernesto K (2006). Continuous System Simulation.

[31] Ruppert M G, Moheimani S O R (2016). Beilstein J Nanotechnol.

